# Overexpression of Apolipoprotein A-I Alleviates Insulin Resistance in MASLD Mice Through the PPARα Pathway

**DOI:** 10.3390/ijms26031051

**Published:** 2025-01-26

**Authors:** Yifan Wang, Yudian Zhang, Yutong Wang

**Affiliations:** Municipal Laboratory for Liver Protection and Regulation of Regeneration, Department of Cell Biology, School of Basic Medical Sciences, Capital Medical University, Beijing 100069, China; wyf@mail.ccmu.edu.cn

**Keywords:** apolipoprotein A-I, insulin resistance, peroxisome proliferator-activated receptor α, metabolic dysfunction-associated steatotic liver disease

## Abstract

Insulin resistance (IR) is one of the important causes of metabolic dysfunction-associated steatotic liver disease (MASLD). Apolipoprotein A-I (apoA-I) is secreted primarily by hepatocytes and plays an essential role in reverse cholesterol transport. Our previous studies revealed that apoA-I can mitigate the progression of metabolic dysfunction-associated steatohepatitis (MASH). However, there is no clear evidence to explain the relationship between apoA-I and IR. Here, we investigated the effects of apoA-I overexpression on IR in both HepG2 cells and mice. In vitro experiment results revealed that apoA-I overexpression can promote cellular glucose uptake in oleic acid-induced IR in HepG2 cells. High-fat, high-cholesterol, and high-fructose diets were used to induce IR in mice. The results showed that apoA-I overexpression improved glucose tolerance, reduced serum insulin levels, and ameliorated IR in diet-induced MASLD mice. Moreover, apoA-I promoted the expression of peroxisome proliferator-activated receptor α (PPARα) in the nucleus both in vitro and in vivo. In conclusion, apoA-I could alleviate MASLD by reducing IR in mice and might exert this effect through the PPARα pathway.

## 1. Introduction

Jurgen Ludwig proposed the concept of non-alcoholic fatty liver disease (NAFLD) in 1980 to describe fatty degeneration of the liver in the absence of alcohol consumption [1]. This concept was revised in 2023 and changed to metabolic dysfunction-associated steatotic liver disease (MASLD) [2]. Similarly, non-alcoholic steatohepatitis (NASH) changed to metabolic dysfunction-associated steatohepatitis (MASH). MASLD is a global health problem, and its increasing prevalence is causing mounting societal economic burden [3]. MASLD can progress to MASH, which is characterised by the ballooning degeneration of hepatocytes and inflammation. MASH increases the risk of end-stage liver disease such as cirrhosis and hepatocellular carcinoma (HCC) [4,5]. The main target organs of insulin include the liver, muscle, and adipose tissue. As the largest digestive organ of the human body, the liver is also the centre of energy metabolism. Insulin resistance (IR) is characterised by hyperinsulinemia, which may promote the pathogenesis of MASLD [6]. A small cross-sectional study showed that liver steatosis is associated with an impaired insulin response in the liver, skeletal muscle, and adipose tissue, both in lean people and in non-diabetic obese people [7,8]. Moreover, when mice are fed with high-fat diet for one week, systemic IR can be detected by a hyperinsulinemic–euglycemic clamp, which is due to hepatic IR [9]. A decrease in insulin sensitivity occurs prior to the stage of hepatocyte steatosis. Therefore, the inhibition of IR maybe an effective treatment for MASLD.

Peroxisome proliferator-activated receptors (PPARs) are nuclear hormone receptors activated by fatty acids. There are three subtypes, namely, PPARα, PPARβ, and PPARγ [10]. In the liver, PPARα is the main subtype, and plays important roles in promoting cellular uptake, esterification, and transport of fatty acids as well as regulating the expression of genes related to lipid metabolism [11,12,13]. Hepatic PPARα mRNA expression decreases with increasing severity of MASH [14]. Notably, liver-specific PPARα knockout mice are more susceptible to high-fat-diet-induced steatohepatitis [15]. Currently, however, no drugs targeting PPARα have been approved for the treatment of MASH. Therefore, finding safer and more effective ways to activate PPARα is highly important for the development of new drugs or treatment options for MASH.

In the liver, apolipoprotein A-I (apoA-I) is secreted by hepatocytes and participates in reverse cholesterol transport (RCT) [16]. In addition, apoA-I also functions as an anti-inflammatory, antioxidant, and antibacterial agent [17]. Our previous studies revealed that apoA-I expression reduced lipid accumulation in HepG2 cells treated with oleic acid (OA) [18]. The specific molecular mechanisms responsible involve the ability of apoA-I to suppress endoplasmic reticulum stress, inhibit autophagy, and reduce oxidative stress in hepatocytes [19,20,21]. Moreover, apoA-I can promote glucose uptake in skeletal muscle cells [22], whereas IR is characterised by a reduced ability to take up glucose. However, there is no evidence to support the relationship between apoA-I and the earlier stage of MASLD, i.e., the IR stage. Our study demonstrated that apoA-I regulates hepatic lipid metabolism through the PPARα pathway and alleviates IR in mice.

## 2. Results

### 2.1. ApoA-I Alleviates OA-Induced IR in HepG2 Cells

**Figure 1:**
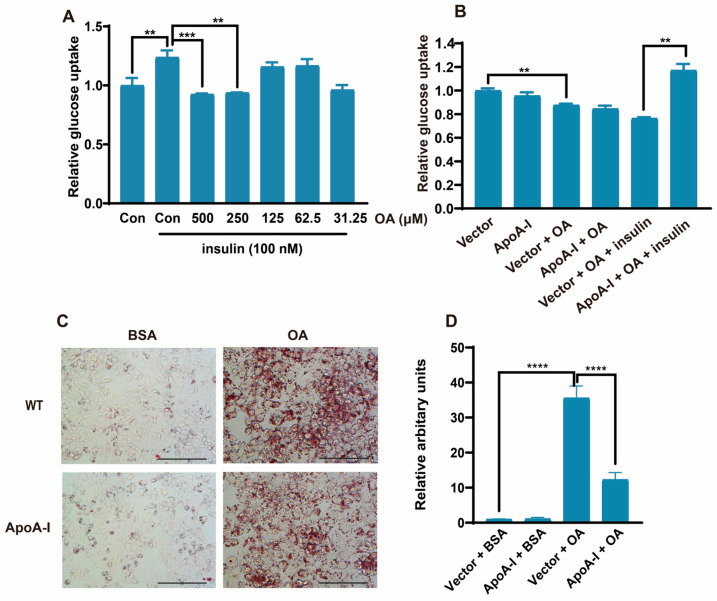
ApoA-I alleviates OA-induced insulin resistance and lipid accumulation. (**A**) Glucose uptake at different concentrations of OA. (**B**) HepG2 cells were transfected with either the pcDNA3.0 or pcDNA3.0/apoA-I plasmid. The transfected cells were incubated for 24 h in DMEM supplemented with 5% BSA in the presence or absence of 500 μM OA. The level of glucose uptake was measured via bioluminescence assay after 20 min of insulin treatment. (**C**) HepG2 cells were transfected with either the pcDNA3.0/apoA-I plasmid. The transfected cells were incubated for 24 h in DMEM supplemented with 5% BSA in the presence or absence of 500 μM OA. After 24 h, oil red O was added to detect lipid accumulation in the cells. Scale bar, 200 μm. (**D**) Oil red O quantification. The data are reported as the means ± SEMs, *n* = 3. ** *p* < 0.01, *** *p* < 0.001, and **** *p* < 0.0001.

OA induces lipid accumulation in HepG2 cells, which is a common in vitro MASLD model [23]. To investigate whether a gradient concentration of OA can induce IR in cells, HepG2 cells were pretreated with different concentrations of OA for 24 h, and then the medium was replaced with a glucose-free medium for 4 h. Furthermore, 100 nM of insulin was added to the glucose-free medium, and the mixture was incubated for 15 min at 37 °C. A glucose Uptake-Glo^TM^ Assay Kit was used to measure the glucose uptake. The results revealed that both 250 μM and 500 μM OA can reduce cellular glucose uptake levels (Figure 1A), and 500 μM OA was selected for subsequent experiments. To determine whether apoA-I could promote glucose uptake in cells, HepG2 cells were transfected with an apoA-I-expressing or control plasmid for 24 h and then treated with or without 500 μM OA for 24 h. The results revealed that apoA-I overexpression had no effect on cellular glucose uptake in the absence or presence of OA. However, when OA-treated cells were stimulated with insulin, apoA-I overexpression significantly increased cellular glucose uptake compared with that in the vector group (Figure 1B). These results indicated that apoA-I promoted glucose uptake in HepG2 cells in an insulin-dependent manner and alleviated OA-induced IR in HepG2 cells.

Moreover, we investigated the effect of apoA-I on lipid accumulation. HepG2 cells were transfected with an apoA-I-expressing or control plasmid for 24 h and then induced with 500 μM OA for 6 h. Oil red O staining was used to detect lipid accumulation after 24 h. The results revealed that apoA-I alleviated OA-induced lipid accumulation (Figure 1C,D). The above results showed that apoA-I could alleviate OA-induced insulin resistance and lipid accumulation.

### 2.2. ApoA-I Promotes the Expression and Transcriptional Activity of PPARα

**Figure 2:**
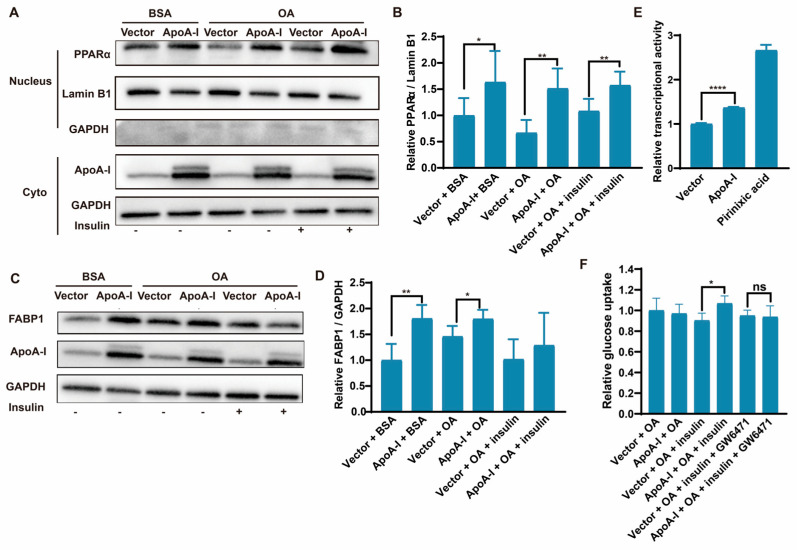
ApoA-I promotes the expression and transcriptional activity of PPARα. Isolation of cytoplasmic and nuclear proteins. (**A**,**B**) Cytoplasmic and nuclear PPARα were detected and quantified with PPARα antibodies. (**C**,**D**) Total cellular protein was isolated and analysed for FABP1 by Western blotting. (**E**) HEK293T cells were transfected with either the pcDNA3.0/apoA-I plasmid. After 24 h of transfection, the positive control group was treated with pirinixic acid for 24 h. PPARα transcriptional activity was detected via a luciferase reporter assay. (**F**) HepG2 cells were transfected with either the pcDNA3.0 or pcDNA3.0/apoA-I plasmid. The transfected cells were incubated for 24 h in DMEM supplemented with 5% BSA in the absence of 500 μM OA and/or 1 μM PPARα antagonists GW6471. The data are reported as the means ± SEMs, *n* = 3, * *p* < 0.05, ** *p* < 0.01, **** *p* < 0.0001, and ns: non-significant.

Previous studies have shown that apoA-I can reduce palmitic acid-induced lipid accumulation in HepG2 cells [20]. PPARα plays an important role in the regulation of liver lipid homeostasis and the development of MASH [24]. Its agonists can promote the expression of apoA-I [25]. However, there is no evidence showing the effect of apoA-I on PPARα. To explore the effects of apoA-I on PPARα, we isolated proteins from the nuclear and cytoplasmic compartments to detect the expression of PPARα in apoA-I-overexpressing HepG2 cells. Compared with the control, apoA-I overexpression significantly increased the expression of PPARα with or without OA or insulin (Figure 2A,B). Moreover, except for in insulin-treated cells, the effect of apoA-I overexpression on fatty acid binding protein 1 (FABP1), which is a downstream gene regulated by PPARα, was a similar pattern to that of PPARα (Figure 2C,D). To explore the mechanism by which apoA-I promotes PPARα protein expression, we transfected the PPRE-TK-luciferase reporter gene into HEK293T cells with either the PPARα, RXRα, or apoA-I expression vector or the control vector. As a result, the expression of apoA-I increased the transcriptional activity of PPARα (Figure 2E). Meanwhile, we treated the cells with the PPARα antagonist GW6471 to detect glucose uptake. The results showed that GW6471 abolished the effect of apoA-I in promoting glucose uptake in cells in an insulin-dependent manner (Figure 2F). In general, apoA-I can not only promote glucose uptake in an insulin-dependent manner but also increase PPARα protein expression by increasing the transcriptional activity of PPARα. Therefore, apoA-I might alleviate OA-induced IR in HepG2 cells through the PPARα signalling pathway.

### 2.3. ApoA-I Attenuates IR in MASLD Mice

**Figure 3:**
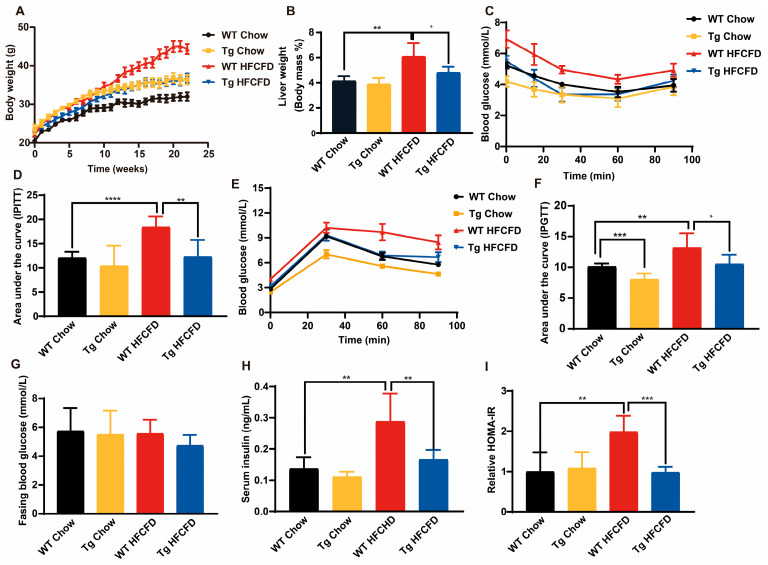
ApoA-I attenuates insulin resistance in HFCFD mice. (**A**) Body weights of the mice in each group. (**B**) Liver index of the mice in each group. (**C**) Intraperitoneal insulin tolerance test (IPITT). (**D**) The area under the curve (AUC). (**E**) Glucose tolerance test (IPGTT) of mice. (**F**) The area under the curve (AUC). (**G**) Fasting blood glucose levels of the mice. (**H**) Fasting serum insulin levels of the mice. (**I**) Homeostasis model of insulin resistance (HOMA-IR). The data are reported as the means ± SEMs, *n* = 5–7, * *p* < 0.05, ** *p* < 0.01, *** *p* < 0.001, and **** *p* < 0.0001.

To explore the effect of apoA-I on IR in vivo, C57BL/6J wild-type (WT) and apoA-I transgenic mice (Tg) were fed a chow diet or a high-fat, high-cholesterol, and high-fructose diet (HFCFD) for 22 weeks. After 15 weeks, the body weights of the HFCFD-fed WT mice were significantly greater than those of the chow diet-fed WT mice. However, there was no significant difference in the body weight of the Tg mice between those fed a chow diet and those fed a HFCFD (Figure 3A). Moreover, compared with that of chow diet-fed WT mice, liver weight was also greater in HFCFD-fed WT mice, indicating that liver oedema, hyperemia, or hypertrophy may have occurred. Compared with those of HFCFD-fed WT mice, the body weight and liver weight of HFCFD-fed Tg mice were significantly lower (Figure 3A,B), indicating that the expression of apoA-I may alleviate HFCFD-induced weight gain and liver oedema or hypertrophy. To assess insulin sensitivity and the ability to control blood glucose in HFCFD-fed mice, an intraperitoneal insulin tolerance test (IPITT) and an intraperitoneal glucose tolerance test (IPGTT) were carried out. Compared with HFCFD-fed WT mice, HFCFD-fed Tg mice presented greater insulin sensitivity and better blood glucose control ability, as evidenced by the reduced area under the curve (AUC) of the IPGTT and IPITT (Figure 3C–F). The fasting blood glucose levels of the four groups of mice were also measured, and there were no significant differences among them, but the fasting serum insulin levels were increased significantly in HFCFD-fed WT mice (Figure 3G,H). These results suggested that the HFCFD reduced the biological efficiency of insulin, which caused HFCFD-fed WT mice to require increased insulin levels to maintain euglycemia. Compared with those in HFCFD-fed WT mice, serum insulin levels in HFCFD-fed Tg mice were lower (Figure 3H). Moreover, the HOMA-IR results revealed that the level of insulin resistance in HFCFD-fed Tg mice was lower than that in HFCFD-fed WT mice, suggesting that apoA-I could alleviate diet-induced IR (Figure 3I).

### 2.4. ApoA-I Alleviates Diet-Induced Liver Injury and Promotes PPARα Expression

**Figure 4:**
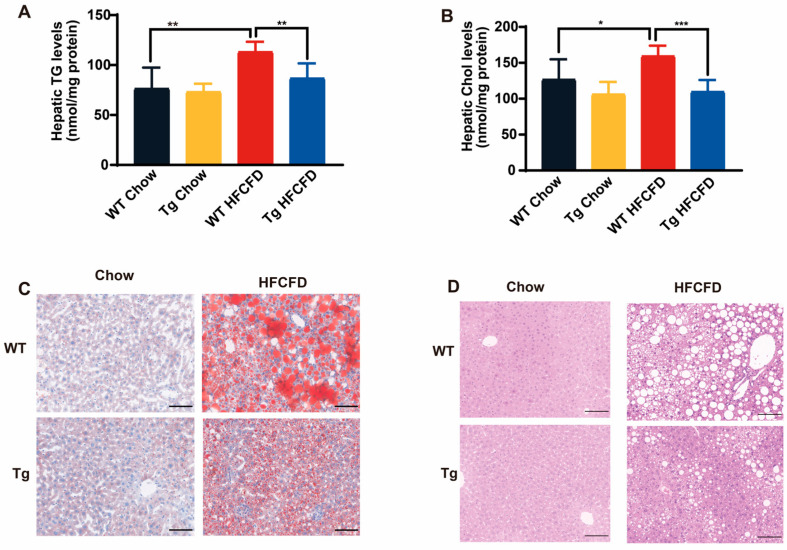
ApoA-I alleviates diet-induced lipid accumulation and liver injury. Hepatic triglyceride (**A**) and cholesterol (**B**) levels. Oil red O (**C**) and H&E (**D**) staining. Scale bar, 100 μm. The data are reported as the means ± SEMs, *n* = 5–7, * *p* < 0.05, ** *p* < 0.01, and *** *p* < 0.001.

**Figure 5:**
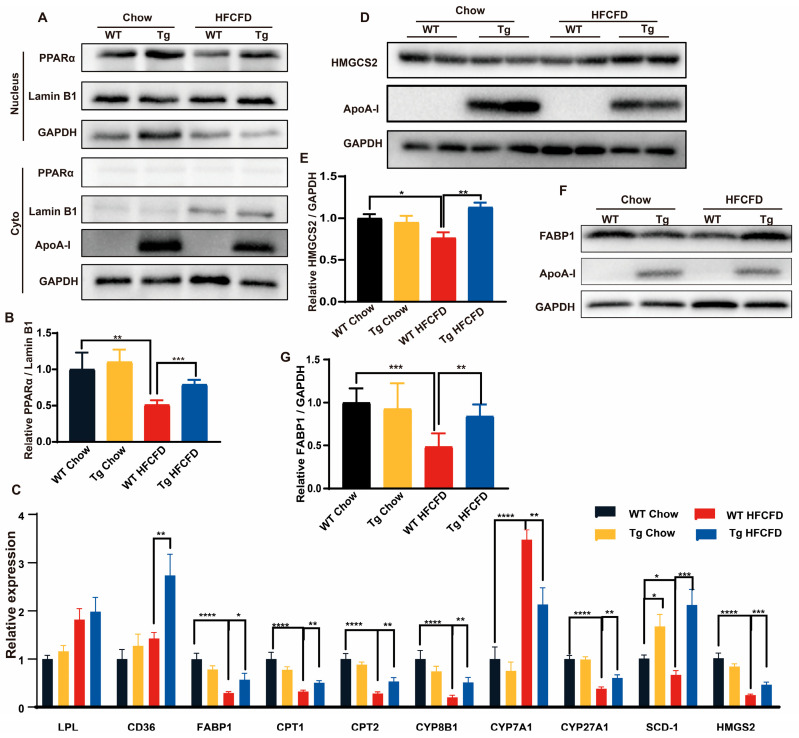
ApoA-I promotes the expression of PPARα. (**A**,**B**) Separation of the nucleus and cytoplasm from liver tissue. Cytoplasmic and nuclear PPARα were detected and quantified with a PPARα antibody. (**C**) mRNA was isolated from the liver, and the mRNA levels of PPARα target genes were quantified via real-time quantitative PCR. (**D**–**G**) Tissue proteins were isolated and analysed for FABP1 and HMGCS2 expression via Western blotting. The data are reported as the means ± SEMs, *n* = 5–7, * *p* < 0.05, ** *p* < 0.01, *** *p* < 0.001, and **** *p* < 0.0001.

The in vitro results above showed that apoA-I might reduce IR in HepG2 cells through the PPARα signalling pathway (Figure 2A,B). To determine whether apoA-I could relieve IR by affecting PPARα in vivo, WT and Tg mice were fed a chow diet or HFCFD for 22 weeks. Compared with HFCFD-fed WT mice, HFCFD-fed Tg mice presented significantly lower alanine aminotransferase (ALT), hepatic total cholesterol (TC), and hepatic triglyceride (TG) levels, with a decreasing trend in aspartate transaminase (AST) levels (Table 1, Figure 4A,B). Moreover, the TGs and TC levels in the serum of the Tg mice were higher than those of the WT mice (Table 1), regardless of whether they were fed a chow diet or a HFCFD, which is consistent with Jackson’s lab’s instructions for the apoA-I transgenic mice. The content of mouse apoA-I in serum showed that chow diet fed Tg mice had lower mouse apoA-I levels compared with chow diet fed WT mice, which is consistent with Jackson’s lab’s instructions. However, compared with chow diet-fed WT mice, HFCFD-fed mice had lower mouse apoA-I levels. The result suggested that HFCFD reduced the serum apoA-I levels. Meanwhile, under the condition of HFCFD, the serum content of free fatty acids in Tg mice was higher (Table 1), indicating that apoA-I can transfer lipids produced in the liver to the peripheral system, which is conducive to alleviating MASLD. Oil red O staining and H&E staining revealed a reduction in hepatic lipid droplets and inflammatory infiltration in HFCFD-fed Tg mice (Figure 4C,D). These results suggested that apoA-I could alleviate diet-induced lipid accumulation and liver injury. In vitro experiments revealed that apoA-I promoted the expression of PPARα in the nucleus (Figure 2A,B). Thus, the nucleus and cytoplasm of liver tissue were separated to detect the protein levels of PPARα. Nuclear PPARα levels were significantly increased in HFCFD-fed Tg mice (Figure 5A,B). To further detect the effect of apoA-I on hepatic PPARα, the mRNA levels of PPARα-regulated downstream genes were measured via qPCR. The results showed that apoA-I could increase the mRNA levels of several downstream genes of PPARα, such as cluster of differentiation 36 (CD36), FABP1, carnitine palmitoyl transferase 1 (CPT1), CPT2, Cytochrome P450 family 8 subfamily B member 1 (CYP8B1), Cytochrome P450 family 27 subfamily A member 1 (CYP27A1), stearoy-CoA desaturase (SCD1), and 3-hydroxy-3-methylglutaryl-CoA synthase 2 (HMGCS2). These results confirmed that apoA-I could promote the transcriptional activity of PPARα in vivo (Figure 5C). The immunoblotting results revealed that the protein expression levels of FABP1 and HMGCS2 were also increased by apoA-I expression. In summary, apoA-I overexpression could promote the expression of PPARα and affected its transcriptional activity in vivo. These results also demonstrated that apoA-I might attenuate IR in MASLD mice through the PPARα pathway.

## 3. Discussion

With an estimated global incidence of approximately 25.2%, MASLD is the most common chronic liver disease in clinical practice [26]. Patients with MASLD usually have several metabolic conditions such as obesity, type 2 diabetes, and hyperlipidaemia [27,28]. However, there are still no approved drugs to treat MASLD. Therefore, the development of new drugs for the treatment of MASLD is urgently needed. ApoA-I is secreted by the liver and intestine and is assembled into high-density lipoprotein (HDL) [29,30,31]. Our study elaborated that apoA-I can alleviate diet-induced IR and alleviate MASLD by promoting the PPARα signalling pathway.

PPARα is a nuclear transcriptional regulator that performs important biological functions by inducing the transcription of downstream target genes [32]. PPARα is the main protein involved in hepatic β-oxidation [33]. It has been reported that activation of PPARα can increase the transcription level of apoA-I [24]. However, it has not been reported whether apoA-I can affect PPARα. Our study demonstrated that apoA-I can increase the transcriptional activity of PPARα in the liver and alleviate lipid accumulation in hepatocytes. However, PPARα expression in the intestine will aggravates the development of MASH [34]. Approximately 30% of the apoA-I in the body is produced in the small intestine. Thus, further study is needed to determine the relationship between intestinal apoA-I and PPARα. At present, PPARα agonists have been used in phase III clinical trials. Compared with PPARα agonist alone, apoA-I has the advantages of not only relieving insulin resistance and lipid accumulation but also improving atherosclerosis.

In our studies, apoA-I promoted the expression of PPARα to alleviate IR. However, the mechanism by which apoA-I promotes the expression of PPARα is not clear. Adipose tissue is not only the largest endocrine organ in the body but also an important target organ of insulin [35]. Adipose tissue can secrete bioactive circulating mediators such as adiponectin, leptin, resistin, and chemotactic proteins, which were collectively known as adipokines [36,37]. Adiponectin secreted by adipose tissue can improve insulin sensitivity [38]. Adiponectin receptor 1 (AdipoR1) and adiponectin receptor 2 (AdipoR2) are expressed in the liver. Some studies have shown that adenovirus-mediated AdipoR2 expression can increase the PPARα signal pathway in LEPR-/- mice [39]. MASLD is often accompanied by obesity. In the case of long-term overnutrition, adipocytes continue to proliferate and hypertrophy to carry more lipids. This process can easily induce inflammation, cause adipose tissue dysfunction, and subsequently lead to reduced secretion of adiponectin. ApoA-I has anti-inflammatory effects. Therefore, we speculate that apoA-I may promote the activation of AdipoR2 on the surface of the liver and promote the expression of PPARα by restoring adiponectin secretion in adipose tissue. Further experiments are needed to support this hypothesis.

In conclusion, our present study demonstrated that apoA-I overexpression could regulate the expression and transcriptional activity of PPARα in the liver, thereby reducing the IR level in MASLD mice. These findings suggest that apoA-I may be a potential target for alleviating IR and may play a therapeutic role in MASLD.

## 4. Materials and Methods

### 4.1. Animal Studies

ApoA-I transgenic (Tg) mice (001927), carrying human apoA-I gene, were purchased from Jackson Laboratory and housed in the Department of Laboratory Animal Science at Capital Medical University. C57BL/6J wild-type (WT) mice were purchased from Vital River Laboratory. All animal experiments were approved by the animal ethics committee of Capital Medical University (AEEI-2024-138). Male C57BL/6J Tg and WT mice aged 6–8 weeks were fed a standard chow or a high-fat (40%), high-cholesterol (2%), and high-fructose (20%) diet (Research Diets, New Brunswick, NJ, USA) for 22 weeks. The body weights of the mice were recorded every week.

### 4.2. Intraperitoneal Glucose Tolerance Test (IPGTT)

The mice were fasted for 12 h before the experiment. A 20% glucose solution was prepared and injected intraperitoneally according to body weight (2 mg/kg). The blood glucose levels of each mouse were measured at 0, 30, 60, and 90 min after the injection. A line chart was drawn, and the area under the curve was calculated.

### 4.3. Intraperitoneal Insulin Tolerance Test (IPITT)

After fasting for 6 h, the mice were given an intraperitoneal injection of insulin (Novolin R, Novo Nordisk, Tianjin, China) solution (0.5 U/kg). The blood glucose levels of the mice at different times (0, 15, 30, 60, and 90 min) were measured after the injection. A line chart was drawn, and the area under the curve was calculated.

### 4.4. Morphological Analysis

After the mice were euthanised, the livers were harvested and fixed in 4% paraformaldehyde. After gradient dehydration, the fixed tissue was embedded in paraffin. Five-micrometer-thick sections were obtained from the paraffin-embedded tissues for haematoxylin and eosin (H&E) staining. The fixed tissue was embedded with optimal cutting temperature (OCT) compound, and 10 μm-thick sections were obtained for oil red O staining.

### 4.5. Biochemical Analysis

Blood was collected from the hearts of the mice. The samples were left at room temperature for 1 h and then centrifuged at 3000 rpm for 15 min to collect the supernatant. Serum glucose, alanine aminotransferase (ALT), aspartate aminotransferase (AST), triglycerides (TGs), and cholesterol (TC) levels were measured via an automatic biochemical analyser (Rayto Life and Analytical Sciences, Shenzhen, China). Fasting serum insulin was measured using ELISA kit (ALPCO, Westlake, OH, USA). Hepatic TGs were measured using triglyceride quantification kit (Applygen Technologies, Beijing, China). Hepatic total TC levels were measured using total cholesterol quantification kit (Applygen Technologies, Beijing, China). Free fatty acid (FFA) levels were measured using non-esterified free fatty acids assay kit (Nanjing Jiancheng, Nanjing, China). Serum human apoA-I levels were measured using ELISA kit (Cusabio, Wuhan, China). Serum mouse apoA-I levels were measured using ELISA kit (Elabscience, Wuhan, China).

### 4.6. Cell Culture

HepG2 cells were purchased from ATCC. HepG2 cells were incubated in high-glucose Dulbecco’s modified Eagle’s medium containing 10% FBS and 1% penicillin and streptomycin. HepG2 cells were transfected with either the pcDNA3.0/apoA-I or pcDNA3.0/vector plasmid in accordance with the manufacturer’s protocol. After transfection, the cell culture medium was replaced with 5 mg/mL BSA in the absence or presence of 500 μM OA to induce IR.

### 4.7. Glucose Uptake Analysis

HepG2 cells were transfected with either the pcDNA3.0/apoA-I or pcDNA3.0/vector plasmid in accordance with the manufacturer’s protocol. After transfection, the cell culture medium was replaced with 5 mg/mL BSA in the absence or presence of 500 μM OA. After the pretreatment of HepG2 cells, the medium was replaced with glucose-free medium for 4 h. Furthermore, 100 nM insulin (Procell, Wuhan, China) was added to the glucose-free medium, and the mixture was incubated for 15 min at 37 °C. A glucose Uptake-Glo™ Assay Kit (Promega, Madison, WI, USA) was used to measure glucose uptake. The fluorescence intensity was measured via a multi-functional microplate detector (TECAN, Männedorf, Switzerland).

### 4.8. Luciferase Reporter Analysis

The PPRE-TK-luciferase reporter gene was transfected into HEK293T cells with PPARα-, RXRα- and apoA-I-expressing plasmids or vector plasmids. After 6 h, pirinixic acid (Selleck, Shanghai, China) was added to the positive control group. After 48 h, the cells were collected for detection. Luciferase assays were performed with the Dual Luciferase Reporter Gene Assay Kit (Yesen Biotechnology, Shanghai, China). Luciferase activity was normalised to Renilla luciferase activity, which was used as an internal control.

### 4.9. Western Blot Analysis

Nuclear and cytoplasmic proteins from HepG2 cells and liver tissues were separated via the NE-PER^TM^ Nuclear and Cytoplasmic Extraction Reagents Kit (Thermo Fisher, Waltham, MA, USA). The protein concentration was determined with a Pierce^TM^ BCA protein assay kit (Thermo Fisher, Waltham, MA, USA). Equal amounts of protein were resolved by SDS-PAGE and transferred to polyvinylidene fluoride membranes. The membrane was subsequently incubated in TBST with 5% skim milk at room temperature for 1 h, followed by incubation overnight at 4 °C with primary antibodies against PPARα (Abcam, Cambridge, UK), apoA-I (Pro-teintech, Rosemont, IL, USA), GAPDH (Kangcheng Biotech, Chengdu, China), Lamin B1 (Proteintech, Rosemont, IL, USA), and Fabp1 (Proteintech, Rosemont, IL, USA). The results were visualised via a ChemiDoc XRS+ system (Bio-Rad Laboratories, Hercules, CA, USA). The greyscale values of the protein bands were calculated via ImageJ version 1.4.3.67 (National Institutes of Health, Bethesda, MD, USA).

### 4.10. RNA Extraction and Reverse Transcription

Total RNA from liver tissues was extracted by the TRIzol reagent. One microgram of total RNA was used for reverse transcription to produce cDNA with HiScript IV RT SuperMix (Vazyme, Nanjing, China) for real-time quantitative PCR (qPCR). qPCR was performed with a Biosystems QuantStudio 5 system (Thermo Fisher, Waltham, MA, USA). GAPDH was used to correct for each sample, and the fold changes relative to the control group were used to determine the final results. The primer sequences are shown in Table 2.

### 4.11. Statistical Analysis

The results of multiple experiments are presented as the mean ± SEM. The statistical significance between two groups was determined via two-tailed *t* tests or one-way ANOVA via GraphPad Prism version 8.0.1. Differences were considered significant if the *p* value was <0.05.

## Figures and Tables

**Table 1:** Serological indicators of four groups of mice.

	WT Chow	Tg Chow	WT HFCFD	Tg HFCFD
ALT (U/L)	44.92 ± 2.81	63.55 ± 6.6	474.5 ± 23.10 ****	108.7 ± 24.6 ^####^
AST (U/L)	171.6 ± 23.63	156.1 ± 24.03	343.3 ± 55.25 *	219.0 ± 45.09
TG (mmol/L)	0.73 ± 0.08	1.15 ± 0.14 ^+^	0.62 ± 0.07	1.21 ± 0.12 ^##^
Cho (mmol/L)	2.43 ± 0.16	6.07 ± 0.25 ^++++^	4.63 ± 0.11 ****	7.50 ± 0.62 ^###^
FFA (mmol/L)	0.70 ± 0.05	0.69 ± 0.04	0.89 ± 0.04 **	1.41 ± 0.13 ^##^
Human apoA-I (mg/mL)	0	36.99 ± 4.67	0	32.21 ± 4.33
Mouse apoA-I (ng/mL)	138.2 ± 9.826	63.43 ± 7.866 ^+++^	62.83 ± 9.777 ***	68.04 ± 15.81

The data were reported as the means ± SEMs, *n* = 5–7, ^#^ WT HFCFD vs. Tg HFCFD, ^##^ *p*  <  0.01, ^###^ *p*  <  0.001 and ^####^ *p* < 0.0001, ^+^ WT Chow vs. Tg Chow, ^+^ *p*  <  0.05, ^+++^ *p*  <  0.001 and ^++++^ *p* < 0.0001, * WT Chow vs. WT HFCFD, * *p*  <  0.05, ** *p*  <  0.01, *** *p*  <  0.001 and **** *p* < 0.0001.

**Table 2:** Primer sequences for qPCR.

Genes	Forward Primers (5′ to 3′)	Reverse Primers (5′ to 3′)
*LPL*	GGGAGTTTGGCTCCAGAGTTT	TGTGTCTTCAGGGGTCCTTAG
*CD36*	AAGCTATTGCGACATGATT	GATCCGAACACAGCGTAGAT
*FABP1*	ATGAACTTCTCCGGCAAGTACC	CTGACACCCCCTTGATGTCC
*CPT1*	CACCAACGGGCTCATCTTCTA	CAAAATGACCTAGCCTTCTATCGAA
*CPT2*	AGCCTACCTGGTCAATGCATATC	GGGTTTGGGTATACGAGTTGAATT
*CYP8B1*	TTGCAAATGCTGCCTCAACC	TAACAGTCGCACACATGGCT
*CYP7A1*	TTCTGCGAAGGCATTTGGAC	AGCATCTCCCTGGAGGGTTT
*CYP27A1*	CAGGAGGGCAAGTACCCAAT	CATTGCTCTCCTTGTGCGATG
*SCD1*	TTCTTGCGATACACTCTGGTGC	CGGGATTGAATGTTCTTGTCGT
*HMGCS2*	GAAGAGAGCGATGCAGGAAAC	GTCCACATATTGGGCTGGAAA

## Data Availability

Data is contained within the article and Supplementary Materials.

## Supplementary Materials

The supporting information can be downloaded at: https://www.mdpi.com/article/10.3390/ijms26031051/s1.
