# Supplementary material for: Overexpression of Apolipoprotein A-I Alleviates Insulin Resistance in MASLD Mice Through the PPARα Pathway
Source: Int J Mol Sci. 2025 Jan 26;26(3):1051. doi: 10.3390/ijms26031051 (PMC11817368; doi:10.3390/ijms26031051)
Supplement: Supplementary file 1 [file ijms-26-01051-s001.zip › ijms-3360837-supplementary.pdf]

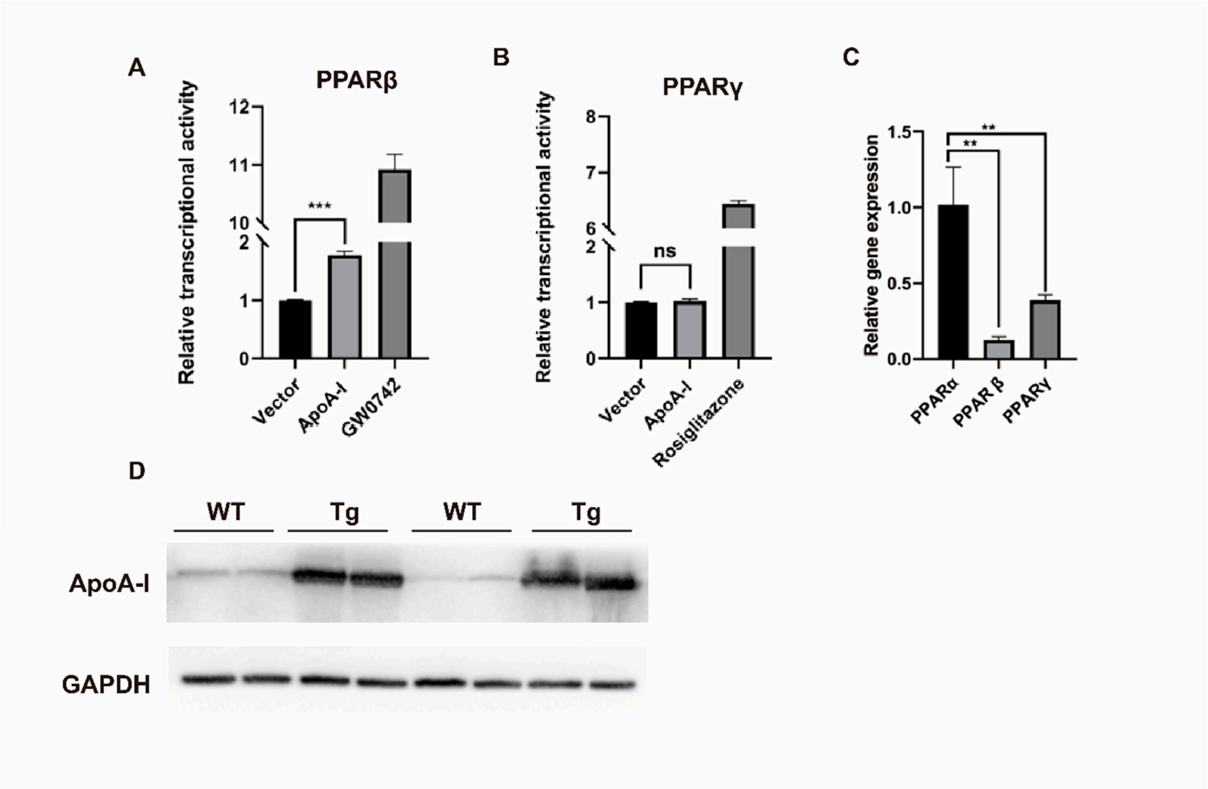

Supplementary Figure

(A)PPRE-TK-luciferase reporter gene was transfected into HEK293T cells with either the PPARβ, RXRα, or apoA-I expression vector or the control vector. After 24 h of transfection, the positive control group was treated with 1 nM GW0742 for 24 h. PPARβ transcriptional activity was detected via a luciferase reporter assay. (B) PPRE-TK-luciferase reporter gene was transfected into HEK293T cells with either the PPARγ, RXRα, or apoA-I expression vector or the control vector. After 24 h of transfection, the positive control group was treated with 42 nM rosiglitazone for 24 h. PPARγ transcriptional activity was detected via a luciferase reporter assay. (C) mRNA was isolated from the HepG2 cells, and the mRNA levels of PPARα, PPARβ, and PPARγ were quantified via real-time quantitative PCR. (D) Tissue proteins were isolated and analysed for apoA-I expression via Western blotting. The data are reported as the means ± SEMs, $n = 3$, ** $p < 0.01$, *** $p < 0.001$, and ns: non-significant.
